# The thriving kids and parents schools project: protocol of an incomplete stepped wedged cluster randomised trial evaluating the effectiveness of a Triple P seminar series

**DOI:** 10.1186/s12889-023-16962-4

**Published:** 2023-10-17

**Authors:** Christopher Boyle, Matthew R. Sanders, Tianyi Ma, Julie Hodges, Kelly-Ann Allen, Vanessa E. Cobham, Igusti Darmawan, Cassandra K. Dittman, Karyn L. Healy, Stevie-Jae Hepburn, Lynda M. MacLeod, Jiachen Teng, Madilyn Trompf

**Affiliations:** 1 School of Education, The University of Adelaide, Adelaide, South Australia 5005 Australia; 2 Parenting and Family Support Centre, The University of Queensland, Queensland, Australia; 3 School of Educational Psychology & Counselling, Monash University, Victoria, Australia; 4 School of Psychology, The University of Queensland, Queensland, Australia; 5 Youth Mental Health Service, Children’s Health Queensland Hospital and Health Service, Brisbane, QLD Australia; 6 School of Health, Medical and Applied Sciences, Central Queensland University, Queensland, Australia; 7 Manna Institute, Central Queensland University, Queensland, Australia; 8 QIMR Berghofer Medical Research Institute, Brisbane, Australia

**Keywords:** Triple P – Positive Parenting Program, Evidence-based Program, Cluster randomised Trial, Parenting seminars, School, Covid-19

## Abstract

**Background:**

The COVID-19 pandemic disrupted the normality of daily life for many children, their families, and schools, resulting in heightened levels of anxiety, depression, social isolation, and loneliness among young people. An integrated public health model of interventions is needed to address the problem and to safeguard the mental health and wellbeing of children. The Triple P – Positive Parenting Program is one system of parenting support with a strong evidence-base and wide international reach. When implemented as a public health approach, Triple P has demonstrated population level positive effects on child wellbeing. This study will be the first large-scale, multi-site randomised controlled trial of a newly developed, low-intensity variant of Triple P, a school-based seminar series, as a response to the impacts of the pandemic.

**Methods:**

The evaluation will employ an Incomplete Batched Stepped Wedge Cluster Randomised Trial Design. At least 300 Australian primary schools, from South Australia, Queensland, and Victoria will be recruited and randomised in three batches. Within each batch, schools will be randomly assigned to either start the intervention immediately or start in six weeks. Parents will be recruited from participating schools. The Triple P seminar series includes three seminars titled: “The Power of Positive Parenting”, “Helping Your Child to Manage Anxiety”, and “Keeping your Child Safe from Bullying”. Parents will complete measures about child wellbeing, parenting, parenting self-regulation and other key intervention targets at baseline, six weeks after baseline, and 12 weeks after baseline. Intervention effectiveness will be evaluated with a Multilevel Piecewise Latent Growth Curve Modelling approach. Data collection is currently underway, and the current phase of the project is anticipated to be completed in January 2024.

**Discussion:**

The findings from this study will extend the current knowledge of the effects of evidence-based parenting support delivered through brief, universally offered, low intensity, school-based parenting seminars in a post pandemic world.

**Trial registration:**

The trial is registered at the Australian New Zealand Clinical Trials Registry (Trial Registration Number: ACTRN12623000852651).

**Supplementary Information:**

The online version contains supplementary material available at 10.1186/s12889-023-16962-4.

The COVID-19 pandemic has had a significant and enduring impact on the mental health of children across the world [1–4]. The lockdowns and school closures undertaken by governments around the world, caused disruption to children’s day-to-day lives and their opportunities for social interaction, which led to increased levels of stress and anxiety for many children [1, 4–7]. Heightened anxiety, irrespective of cause, has a direct of effect on children’s academic performance, wellbeing, and brain development [8]. Various studies have also shown that the mental health effects of the pandemic are reflected in increased levels of depression, social isolation, and loneliness [1, 5–7]. The COVID-19 pandemic has disrupted normality for many children, families, and schools, in ways that might not be completely clear for years to come, suggesting that effective and ongoing interventions and support are required.

There is little doubt that the implementation of various lockdown measures and the urgent move to online learning in schools would have presented many challenges to families and schools. The psychological impact of COVID-19 is likely to be more pronounced in situations where children already had limited support networks or there was poor access to professional support. Some of the direct impact on children of the shutdown of services during the pandemic was the loss of in-person contact with extended family and friends but also loss of access to the emotional and learning support provided in direct interactions with teachers. Beginning schooling online and then the stop-start approach to schools being open or not has led to a difficult time especially for children. These effects are documented in the outcomes of an online survey of 1327 parents and carers of Australian children aged 4 to 17 years. This survey found that 30.5% parents reported their child’s emotional symptoms in the high to very high range and 20.2% of parents indicated that their children were experiencing clinical levels of anxiety symptoms. Parents also reported high to very high levels of conduct problems (26.3%) [4]. Interestingly, a study from the United Kingdom found greater deterioration in the mental health of preadolescent children [2], with the proposal that the loss of face-to-face social interactions in this age group was more impactful than for adolescents who were better able to maintain friendships online. The COVID-19 pandemic has shown that the psychological longer-term consequences may be quite pronounced. From a parental perspective, they were suddenly thrust into an environment when they had to support their child’s learning, at home, for a considerable amount of time. This could be whilst having no idea if they would continue to be employed due to the economic uncertainty and the government having to intervene to contribute towards salaries. The added factor of needing to have a good internet connection coupled with appropriate IT equipment for all to work online could be daunting. There is a myriad of stress points here which dramatically affected how people were able to manage during the pandemic. These issues indicate that there is a need for interventions in various areas such as that of school and family.

The general disruption caused by the pandemic and the actions taken by governments have had far reaching consequences for families. The uncertainty around the impact of COVID-19 and the worry about when the restrictions and dangers may end were exacerbated by changing public health guidelines; this resulted in many parents feeling overwhelmed and concerned about their own mental health and especially that of their children [9]. Westrupp, Bennett [10] compared the responses of 2365 Australian parents during stage 3 of the COVID-19 restrictions (April 2020), to pre-pandemic data. This study found that during the pandemic parents reported significantly higher rates of depression, anxiety, stress and irritability and considerably lower rates of positive emotional expressiveness. More parents also reported drinking four or more days per week compared to prior to the pandemic. These outcomes highlight the adverse effects of the COVID-19 pandemic on parents’ wellbeing, which also has implications for children who may be sensitive to the worries of their parents.

As children returned to school, the ongoing impact of the COVID-19 pandemic manifested in many ways, such as problem behaviours, emotional distress and in challenges in peer interactions both in the class and the wider school environment [11, 12]. The impact of the COVID-19 pandemic on children’s wellbeing has amplified the need for additional mental health supports, which has challenged and overstretched existing individual-level services, with many young people unable to access the help they need [12]. A systematic review of mental health interventions for children and adolescents found that school-based interventions that foster partnerships between teachers, parents and other professionals resulted in decreased levels of anxiety, depression and conduct problems and in an increase in social skills [13, 14]. Westrupp, Bennett [10] also emphasise the importance of improving both child and parent mental health via additional support for parents. The Triple P Positive Parenting Program [15–17] provides a potential model of intervention that can be adapted to provide evidence-based parenting support in a school context.

Given the widespread adverse impact of the COVID-19 pandemic on the mental health and wellbeing of children and their families an integrated public health model of intervention is needed to address the problem. The Triple P Positive Parenting Program [15–17] provides a potential model of intervention that can be adapted to provide evidence-based parenting support in a post pandemic environment. The Triple P multilevel system has five different levels of parenting support. The Level 2 (Triple P Seminar series) seem to be particularly relevant as it is designed as a brief, low intensity intervention that can be delivered universally (either in person or via telehealth), in schools and at low cost.

There is a strong evidence-base for the effectiveness of the original Triple P Seminar program that comprises three 90–120 min seminars, as well as more recent adaptations of seminars for special populations of children. Specifically, four lines of evidence support the efficacy of parenting seminars in improving social, emotional and behavioural problems in children − 1) individual randomised controlled trials evaluating the original three seminars-The Power of Positive Parenting; Raising Confident, competent Children; and Raising Resilience Children in Western Countries [18]; 2) replication studies of Triple P seminars with non-Western populations including in Indonesia [19] and Iran [20, 21]; 3) adaptations of the original universal seminar program to include parents of children with a disability (Stepping Stones Triple P Seminars; 22, 23], parents with concerns relating to children’s anxiety (Fear-Less Triple P Seminar; 24], and parents seeking to promote healthy lifestyles in their children (Lifestyle Triple P Seminars; 25]; and 4) finally, studies that have incorporated Seminars as part of a wider mix of levels of Triple P as part of population rollouts of the Triple P system in large-scale population studies targeting child social, emotional and behavioral problems (e.g., 26, 27, 28] and the prevention of child maltreatment [29]. Overall, this research demonstrated that the Triple P seminars are an effective low-intensity and highly feasible intervention that has positive effects on parenting and a range of child emotional and behavioral outcomes, with several studies demonstrating these improvements are maintained over the longer term [19, 22, 23].

Sanders, Prior [18] demonstrated that Triple P seminars were effective in changing Australian parents’ parenting practices and improving child social, emotional and behavioral problems. This evaluation showed that the seminars were effective in reducing dysfunctional parenting practices and child behavior problems. Bartlett, Sanders [25] evaluated the effects of three Lifestyle Triple P seminars focused on promoting healthy lifestyle in children. There was a significant intervention effect on ineffective parenting, lifestyle-specific and general parenting confidence. Child lifestyle problem behaviors also reduced.

Several studies have demonstrated the effectiveness of parenting seminars. In Indonesia, Sumargi, Sofronoff [19] found that parents who attended Triple P seminars reported a greater decrease in child behavioral problems, dysfunctional parenting practices, parental stress and a greater increase in parenting confidence compared to parents in the waitlist control group at post intervention. The intervention effects were maintained at 6-month follow up for parents in the intervention group. In Korea, Lee, Keown [22] evaluated a Stepping Stones Triple P Seminar series for parents of children with a disability. Significant short-term intervention effects were found for reductions in child behavior and emotional difficulties, and dysfunctional parenting practices. These improvements were maintained 4-months later by the intervention group.

The present study, the Thriving Kids and Parents – Schools Project (TKPSP), extends the available literature on the effects of parenting seminars by testing the effects of a seminar series designed specifically to address the needs of parents and children in a post pandemic environment. Specifically, we aim to evaluate the effects of three seminars – one focusing on general parenting skills (“The Power of Positive Parenting”), one focusing on helping children manage anxiety (“Helping Your Child to Manage their Anxiety”) and the other focussed on improving children’s peer relationships and reducing the risk of being bullied by peers (“Keeping your Child Safe from Bullying”). Each seminar drew on content from more intensive clinical interventions, Triple P Positive Parenting, Fear-Less Triple P and Rsilience Triple P with the following outcomes reported. A meta-analysis involving 16,099 families examined the effects of the Triple P-Positive Parenting Program found significant short and long-term effects were found for children’s social, emotional and behavioral outcomes, parenting practices, parenting satisfaction and efficacy, parental adjustment and parental relationship [16]. A randomised controlled trial of a Fear-Less Triple P, a brief 6-session parent-focused intervention for anxiety disordered children, found that the of the 61 families in the intervention group, the percentage of children free of any anxiety diagnosis was 38.7% (post-treatment), 58.6% (3 month0; 69.2% (6 months) and 84% (12 months) compared to 3.4% in the wait-list condition [30]. In a randomised controlled trial of Resilience Triple P (an intensive family program targeting supportive parenting and child social and emotional skills), that involved 111 families, teachers of children in the intervention group reported greater improvements in peer acceptance and reductions in being bullied compared to the wait-list control group [31].

Based on the strong evidence for the more intensive versions of each of the seminars, we anticipate finding improvements in primary intervention targets, including child social, emotional, and behavioural wellbeing, general parenting practices, and parenting self-efficacy/self-regulation. We also expect to find improvements in secondary outcomes such as child anxiety, depression, family adjustment, and specific parenting practices that facilitate child anxiety. One exploratory aim is examining whether the intervention would strengthen the parent-report quality of the home-school partnership. Improvements are expected to be similar across both start now and start later conditions.

## Methods and analysis

### Funding

This research was funded by the Australian Government Department of Education, Skills and Employment through the Emerging Priorities Program (November 2022 to February 2024).

### Ethics

The study was granted an official ethical approval by the University of Queensland Human Research Ethics Committee (ID: 2022/HE001114). Ethics notification has been accepted by the University of Adelaide Human Research Ethics Secretariat and Legal and Risk Office (ID: 37018), and Monash University (ID: 36385). Before recruiting schools, ethics approval was obtained from the Queensland, South Australian and Victorian Departments of Education. Approval was also obtained from relevant Catholic Dioceses in each of the three states included in this study (i.e., Queensland, South Australia and Victoria).

### Design

The evaluation will employ an Incomplete Batched Stepped Wedge Cluster Randomised Trial Design. As informed by relevant methodological guidelines [32–34], Stepped Wedge Cluster Randomised Trial Design was chosen instead of a more traditional parallel cluster randomised controlled trial for three reasons, Firstly, this is a more feasible randomised trial design given that the randomisation will be conducted at the school level. Due to the lengthy wait required by a parallel cluster randomised trial design, it is anticipated that schools allocated to the waitlist control condition using this design would be less likely to be sufficiently motivated to endorse the program by completing three surveys prior to accessing the seminars. In addition, using the parallel cluster randomised trial design, parents in the control schools are less likely to complete three waves of assessment prior to receiving a low-intensity seminar intervention. Secondly, in the parallel design all schools will receive the intervention at once, while a sequential rollout accommodates capped delivery capacity. An incomplete design was used to minimise the measurement burden [33, 35, 36].

**Fig. 1:**
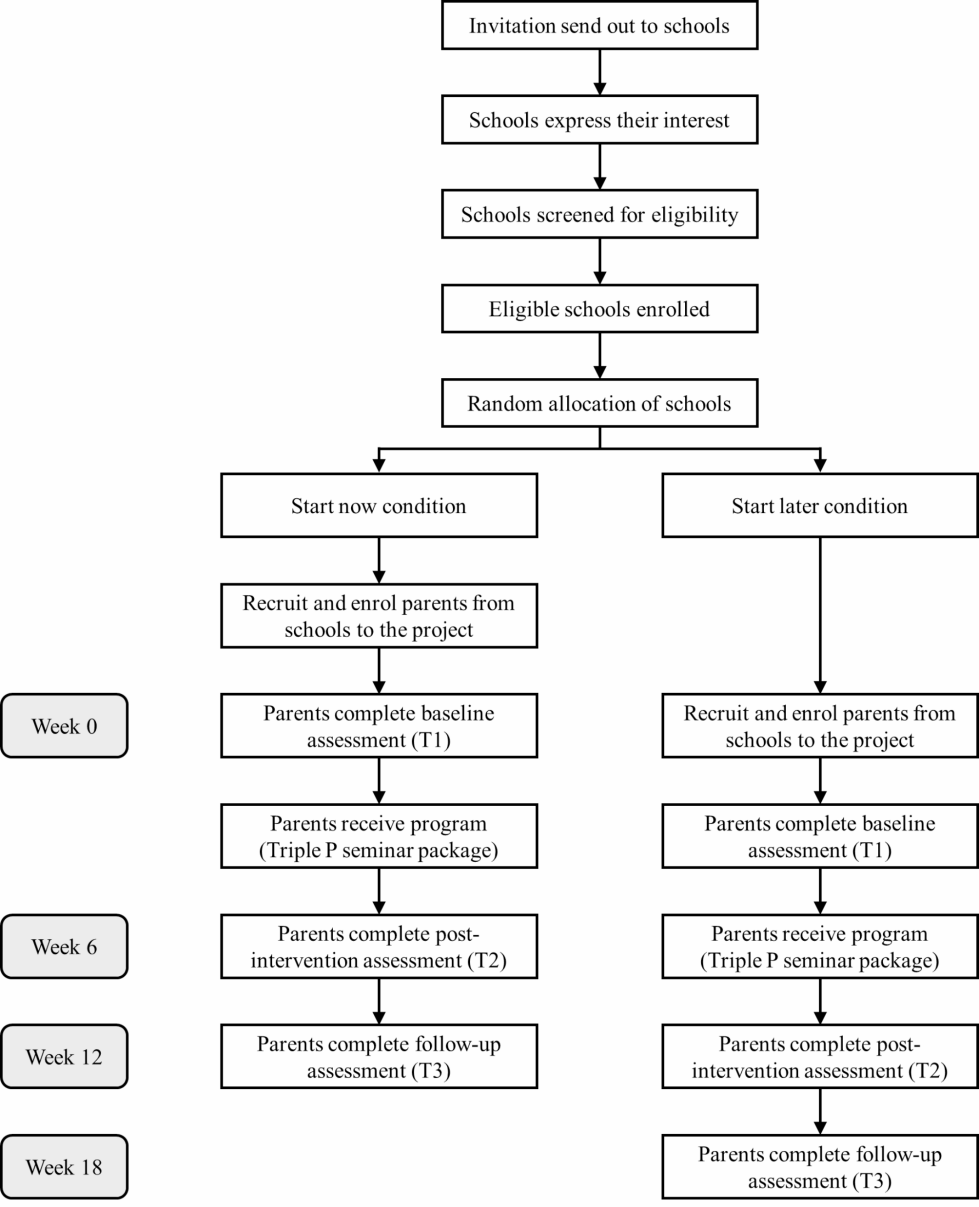
CONSORT Flow Diagram of Study Procedure

A batched component was added to the research design to account for ongoing recruitment of schools [37]. Schools will be recruited in three batches, with approximately 100 schools in each batch. Within each batch, schools will subsequently be randomly allocated to: (1) receiving the seminar package immediately (start now); or (2) receiving seminars 6 weeks later (start later). Each successive batch will start six weeks after the commencement of the previous batch. The evaluation will employ a quantitative online survey method with data collected from all participants before the intervention, six weeks after the initial assessment (Time 2), and 12 weeks after initial assessment (Time 3). For detailed study design and project timeline, please see Table 1. Recruitment and data collection are currently underway and are expected to be completed by January 2024. For detailed study procedure, please see Fig. 1.

### Participants and recruitment

The study will involve at least 300 Australian primary schools, from three Australian states, South Australia, Queensland, and Victoria. Following the receipt of relevant ethical clearance, school leaders will be notified about the Thriving Kids and Parents Schools Project through multiple channels (e.g., emails, principal, and parent associations). Priority will be given to government (state-funded) and Catholic schools and if funding permits, independent schools will subsequently be invited to participate. Initial school engagement will utilise an expression of interest (EOI) approach with interested schools being required to complete an EOI form. Following completion of an EOI, further project information will be provided and processes regarding participation negotiated.

Using promotional materials produced by the project team, participating schools will invite their parent body to register online to participate in the TKPSP seminar series. The promotional materials will be specifically targeting schools’ multiple communication channels with the aim of recruiting as many parents as possible. In order to appeal to a wide audience, parents’ eligibility will be determined using the following broad inclusion criteria: (1) presence in the family of at least one child of primary-school age; (2) interested in information about parenting and child development; (3) able to attend online seminars on three separate occasions. Parents will be required to register online and consent to participate will be collected during the registration process.

Utilising the method introduced by Hemming and colleagues [38, 39], assuming an effect size of 0.30 with a type one error rate of 0.001 and an intra-cluster correlation coefficient of 0.05, engaging 50 schools in each batch with an average of more than 10 parents per school will produce sufficient statistical power (> 0.90).

### Intervention and intervention delivery

The intervention used in the present study consists of three Triple P seminars, which is a universal offer designed for all parents. The seminars are likely to be of particular interest to parents who are generally coping well but having some concerns about aspects of their child’s social or emotional development or have parenting concerns and are seeking further information and support. Each seminar takes about 90 to 120 min, including Question and Answer time. The three interconnected seminars focus on an important set of strategies to help parents support their child’s development and help them thrive now and onwards into adolescence. Seminars in the TKPSP series cover the topics of “*The Power of Positive Parenting*”, “*Helping Your Child to Manage Anxiety*”, and “*Keeping your Child Safe from Bullying*”.

All seminars will be organised by the TKPS project team and delivered by accredited Triple P practitioners via videoconferencing software (i.e., Zoom Webinar). According to the latest data from the Australian Communication and Media Authority [40], more than 99% of all Australian adults have access to the internet, and 93% of Australian adults have internet connection at home. For parents who do not have internet access at home, local arrangements will be made through participating schools to provide internet access, enabling all interested parents to participate. This model has been demonstrated to be effective in a Triple P trial in Los Angles, where the sample contained a high proportion of disadvantaged parents who did not have internet access at home [41]. Towards the conclusion of the TKPS project, and while remaining within the budget, schools will be invited to nominate a staff member to be trained in delivering Triple P seminars. Offering online seminars to parents ensures that as many parents as possible will benefit from the TKSPS seminar series, while the aim of training school staff is to provide an opportunity for ongoing seminar delivery, thus supporting sustained delivery beyond the TKPSP.

### Teachers’ resources

In addition to providing seminar training for a sub-section of interested teachers, resources have been developed for use by teachers from all participating schools. The teacher resource package consists of a brief webinar summarising the content of each of the three parent-seminars. The webinar will be housed on a secure platform and can be accessed by teachers at a time of their choosing and re-visited as needed. Teachers will also be provided with a detailed summary of strategies presented in each of the seminars including examples of how these strategies might be used in the classroom. Interested teachers will also be invited to attend online parent-seminars. The purpose of providing complementary teacher resources for participating schools is to foster a common language and a shared set of strategies to support children’s wellbeing. It is anticipated that providing parents and teachers with complementary information and resources will enhance the partnership between families and schools (home-school partnership) in participating schools. Research has shown both enhanced learning and wellbeing outcomes for children when parents and teachers engage in positive two-way communication (Fantuzzo et al., 2000; Reschly & Christenson, 2012; Smith et al., 2020).

### Randomisation

Following an expression of interest in participating in the project, schools within each batch of schools will be randomly allocated to either the ‘start now’ condition or the ‘start later’ condition. Random allocation will be conducted on an ongoing basis throughout the trial via a process of Minimisation, which aims to achieve the optimal balance of school characteristics between the two study arms throughout the trial. Minimisation is the only acceptable alternative to randomisation in the CONSORT guidelines [42]. Schools will be randomised in a 1:1 ratio, with a random component (*p* = .80), using the *Minirand* Package in *R Studio* [43]. The latest school demographic information will be accessed via the My School website [44]. The following demographic factors will be included in the minimisation process: state (Queensland, South Australia, Victoria), school sector (state, catholic, or independent), school size (enrolment number < 100, 100–499, 500–999, or > 1,000), socio-economic status (the index of community socio-educational advantage [ICSEA]: lowest quarter, second quarter, third quarter, highest quarter), school location (major cities, inner regional, outer reginal, remote, very remote), school type (co-education, single sex). Parent recruitment at each school will begin 2 to 4 weeks prior the intervention rollout at each school.

### Outcomes

A comprehensive set of measures will be administered to track changes in child and family related outcomes over time. Parent-report survey data will be collected online, through the Qualtrics survey platform at baseline (T1), post-intervention (T2; 6 weeks after T1), and follow-up (T3; 12 weeks after T1). At each assessment point, the online survey will take about 15 to 20 min to complete. A prize draw for gift vouchers was introduced to increase the survey response rate at T2 and T3. Participants will not be informed about the introduction of the prize draw prior to the due date of the survey. Data will be stored in Australia on secure university servers. Table 2 displays detailed information about when each measure will be administered. Below is a list of descriptions for each individual measure.

#### Demographic questionnaire

Socio-demographic characteristics will be collected with a modified version of the Family Background Questionnaire (FBQ) [45]. A range of factors will be assessed, such as child age, gender and health, parent age, gender, marital status, education level, and cultural background.

#### Parenting and Family Adjustment Scale (PAFAS)

Parenting practices and family functioning will be assessed using the Parenting and Family Adjustment Scale (PAFAS) [46]. The PAFAS includes two scales – a 18-item Parenting Scale and a 12-item Family Adjustment Scale. The Parenting Scale contains four subscales, namely *Parental Consistency*, *Coercive Parenting*, *Positive Encouragement*, and *Parent-child Relationship* with adequate to good internal consistency (H = 0.70, 0.78, 0.75, 0.85 respectively). The Family Adjustment Scale has three subscales, namely *Parental Adjustment*, *Family Relationships*, and *Parental Teamwork* with good internal consistency (H = 0.87, 0.84, 0.85 respectively). Responses will be rated on a four-point Likert scale ranging from 0 (not true of me at all) to 3 (true of me very much). The total score for each subscale is calculated by summing the responses for each item. Higher scores indicate higher levels of dysfunction. The *Parent Adjustment* subscale can also be used as a stand-alone measure [47].

#### Parenting self-regulation scale (PSRS)

The 12-item Parenting Self-regulation Scale (PSRS) [48] will be used to assess parents’ self-regulatory capacity in their parenting role. The PSRS has a single factor structure and demonstrated excellent internal consistency (α = 0.92). Each item will be rated on a Likert scale from 1 (strongly disagree) to 7 (strongly agree). A total score is calculated by summing all items. Higher scores reflect higher parenting self-regulation.

#### Child Adjustment and parent efficacy scale (CAPES)

Children’s social, emotional, and behavioral difficulties, as well as their parents’ confidence in managing the parenting challenges will be assessed by an extended version of the Child Adjustment and Parent Efficacy Scale (CAPES) [49]. The original CAPES has 27 items to measure the intensity of child emotional and behavioral problems, with a 3-item emotional maladjustment and a 24-item problem behavior subscale. The internal consistency for the total intensity subscale and the subscales ranges from adequate to excellent (α = 0.90, 0.90, and 0.74 respectively). Item ratings are indicated on a 4-point Likert scale of 0 (not true of my child at all) to 3 (true of my child very much). Nineteen items describe negative behavior, and eight reverse-coded items describe positive behavior. Parents are also asked to rate their confidence in managing each of the negative behaviors using a scale of 1 (certain I can’t do it) to 10 (certain I can do it). The sum of the 19 confidence ratings generates the Efficacy scale of the CAPES, which has excellence internal consistency (α = 0.95). In the present study, five items have been added to capture children’s social difficulties with peers, through three negatively worded items (e.g., “*my child has no one to play with*”) and two positively stated items (e.g., “my child has close friendships at school”). Parents are required to provide confidence ratings for each of the negative statements.

#### Partner in Education Survey (PIES)

The Partner in Education Survey (PIES) [50] is a parent-report measure the Home-School Partnership for parents of children ages 5 to 12 years attending primary school. This 18-item measure can be completed by a cross-section of parents from differing backgrounds and education levels and, as such, the comprehension level and readability were considered. The PIES has a Flesch-Kincaid Grade Level of 7.2. This readability statistic means that the survey is able to be understood by someone who has obtained at least grade seven schooling or seven years equivalent education. The PIES contains five sub-scales: Parent-Teacher-Communication, Parent-School Communication, Home Involvement, Parental-School Contribution and Working with the Community with three additional items being retained due to their clinical significance (*I value learning and try to communicate this value to my child*, *I feel comfortable talk* and *My child’s teacher and I have mutual respect for one another*). Parents respond to each item on a 10-point Likert-type response scale ranging from 1 *never/not at all* to 10 *always/very much*. An overall score is calculated by summing the total of all responses with higher scores representing more positive parental perceptions of the Home-School Partnership. The internal consistency of the total scale is excellent (α = 0.92).

#### Parenting an anxious child questionnaire (PACQ)

Parenting an Anxious Child Questionnaire (PACQ) [51] is used to assess parental responses and confidence in managing children’s anxiety. PACQ has 12 items, where 11 items assess parents’ frequency of using each specific parenting strategy and the 12th item assesses parents’ overall confidence in managing children anxiety. Items are rated on a five-point Likert-scale ranging from 0 (Never) to 4 (Always). The sum of item ratings (with three reverse-coded items) is used to calculate the total score.

#### Brief Spence Children’s anxiety scale (BSCAS)

Child anxiety symptoms will be measured with the parent-report version of the Brief Spence Children’s Anxiety Scale (BSCAS) [52], which is an abbreviated version of the Spence Children’s Anxiety Scale – Parent version [53]. The BSCAS is an 8-item, single factor measure with adequate to good internal consistency (α = 0.73-0.82 in different samples). Parents rate their responses on a four-point Likert scale, ranging from 0 (Never) to 3 (Always). A measure score is calculated by summing all the items; higher scores indicate higher levels of child anxiety.

#### Preschool feelings Checklist (PFC)

The Preschool Feelings Checklist (PFC) [54] is a 16-item checklist of children depression symptoms. The PFC contains 16 statements about child mood and depression where parents answer yes/no for each statement. The PFC has acceptable internal consistency (α = 0.76). The sum of scores is used to calculate the PFC score with higher scores suggesting higher levels of paediatric depression.

#### Parent satisfaction survey (PSS)

The Parent Satisfaction Survey (PSS) [55] evaluates parents’ perceptions of the overall quality of the Triple P seminar series. The PSS consists of 10 items capturing different aspects of the seminars. Parents rate their response on a 7-point Likert-scale ranging from 1 (*poor/no, definitely not*) to 7 (*excellent/yes, definitely*). Parents are also asked to provide general comments about the seminar series.

#### Analytic plan

**Fig. 2:**
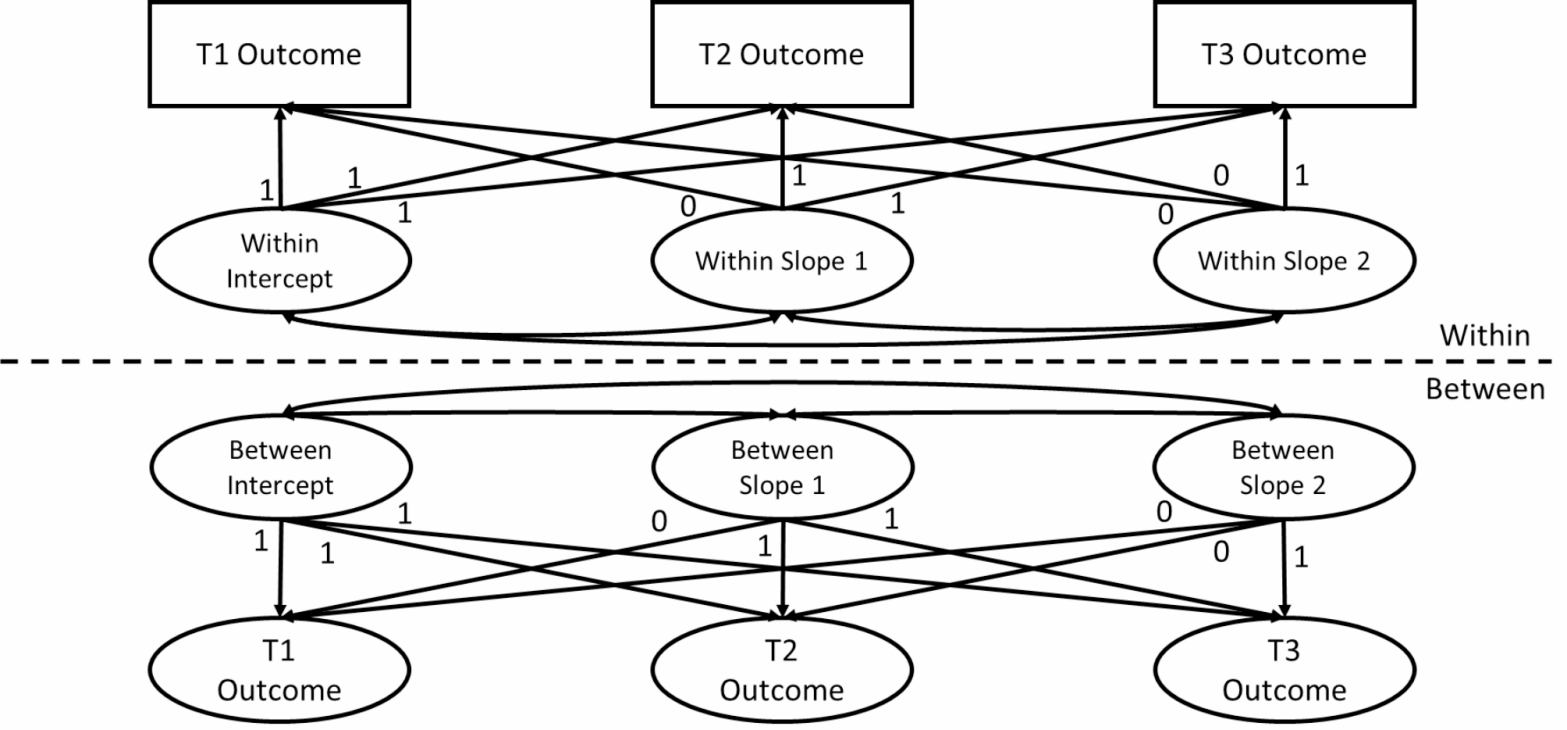
Proposed Multi-level Piecewise Latent Growth Curve Model

Only non-identifiable data will be included in analysis. The patterns of the data missingness will be tested and appropriately addressed prior to the analysis of outcome variables. If the missingness is deemed to be Missing Completely at Random or Missing at Random, Full-information Maximum Likelihood (FIML) estimation or expectation maximisation algorism (EM) will be used to impute the missing data [56]. Following an intention-to-treat protocol, a Piecewise Latent Growth Curve Model (Piecewise LGCM) will be tested for each outcome variable, which allows the estimation of the rate of change between the pre-intervention assessment and the post-intervention assessment and the rate of change between the post-intervention assessment and the follow-up assessment. If the school level variance is substantial due to the nested nature of sample, a multi-level Piecewise LGCM will be used. School and individual level control variables might be included in the model (see Fig. 2 for the proposed model). To examine the differences between conditions, appropriate analytic options for the present study design will include but not limit to multigroup ML-Piecewise LGCM and visual analysis using Brinley Plots [57].

## Discussion

The findings from this study will extend our current knowledge of the effects of evidence-based parenting support delivered through brief, universally offered, low intensity parenting seminars and delivered in school settings. The approach adopted is consistent with the multi-level conceptual model of evidence-based parenting support for educational settings as outlined by Sanders, Healy [58]. The model highlights the unique value of the school setting to help normalize and destigmatize parenting programs and thereby increase parental engagement and widen the reach of parenting programs.

The intervention being tested builds on previous studies showing that a brief three session Triple P seminar series on positive parenting can be effective in changing parenting practices and in improving children’s behavior and adjustment [16, 19, 23]. It extends earlier work by concurrently addressing in the same program, parents’ concerns about their children’s behavior problems, anxiety and peer relationships, particularly school bullying. As the seminar series is a low intensity, brief intervention (Level 2 in the multilevel Triple P system) it is expected that a minority of children and parents with more complex problems may require additional support.

The sample size we intend to recruit will be large enough to examine important moderators of intervention outcomes for both child and parent outcomes including parental completion rates. These moderators include the type of school (state, Catholic, or independent school), the relative social disadvantage of schools, child and parent age and gender, family characteristics (e.g. single parent household), ethnicity, the baseline severity of child social, emotional and behavioral problems, parenting practices and level of program participation such as the number of seminars parents attended. These analyses will help identify the profiles of children and parents who might require early additional support to complete the series and lead to a more nuanced understanding of the reasons for non-completion. As each of the individual seminars are presented as a package and introduced as being relevant to every child, it is likely that some parents would be particularly interested in certain topic and less interested in others. Parents and teachers’ evaluations of the seminars themselves will provide additional end user/consumer feedback to enable the program to adapt and evolve [59, 60].

The interpretation of findings from this study will need to take into account the study’s relative strengths and limitations. Relative strengths include recruiting a large number of schools and parents and the socioeconomic diversity of the schools involved that included state public schools, Catholic Schools and independent schools. The outcome assessments will include reliable, validated and change sensitive assessment tools. The experimental design will enable the program to be sequentially introduced, across the school year, using an incomplete stepped wedge cluster randomised trial design. This variant of the stepped wedge design is particularly useful in evaluating programs in schools. This design involves a systematic replication of intervention effects, with schools servicing as their own controls, rather than relying on randomisation of schools to different conditions e.g., where the intervention is withheld altogether, not available, or only after long delays as in care as usual and waitlist control designs. Traditional RCT’s can be very difficult to implement in regular school settings with differential attrition between intervention and control schools being a particular concern. Another unique feature of the approach will be the inclusion of an intervention component, via a teacher webinar, that will provide guidance to teachers on how to support parents participating in the parenting seminar series. It is widely recognised that improved home-school communication can greatly benefits children’s experience of school, their learning and social and emotional wellbeing, all of which had been adversely affected by COVID-19 [4].

The relative weaknesses of the study include reliance on parents (likely to be disproportionately mothers) as the primary informant for gauging intervention effects. Although parents are the primary target of the intervention and, as such, are crucially important informants about their experience, it is not possible to independently verify parent reported change in their children’s behavior, anxiety or peer relationships. It would have been valuable to plan collection of independent teacher observation data on the child outcomes; however, this is precluded as based on the ethical reasons, teachers were not able to be informed which parents at their school had actually attended the online seminars which were generally scheduled out of school hours.

**Table 1:** Incomplete Batched Stepped Wedge Cluster Randomised Trial Design with Project Timeline

Batch	Step	Time (2023)					
		May	Jun/Jul	Aug	Sep/Oct	Nov	Dec/Jan 2024
		Wk0	Wk6	Wk12	Wk18	Wk24	Wk30
1	1	○	●	●			
	2		○	●	●		
2	1		○	●	●		
	2			○	●	●	
3	1			○	●	●	
	2				○	●	●
School Recruitment (N)		100	100	100			
○ Baseline assessment			● Post-intervention assessment			Intervention delivery	

*Note*. Randomisation will determine whether the school will start in step 1 or 2 at each batch

**Table 2:** Measures Administration Schedule

Measure name	Item *N*	T1	T2	T3
Demographic Questionnaire		●		
Parenting and Family Adjustment Scale (PAFAS)	30	●	●	●
Parenting Self-regulation Scale (PSRS)	12	●	●	●
Child Adjustment and Parent Efficacy Scale (CAPES)	32	●	●	●
Partner in Education Survey (PIES)	18	●	●	●
Parenting an Anxious Child Scale (PAAC)	12	●	●	●
Brief Spence Children’s Anxiety Scale (BSCAS)	8	●	●	●
Preschool Feelings Checklist (PFC)	20	●	●	●
Parent Satisfaction Questionnaire (PSQ)	10		●	

### Electronic supplementary material

Below is the link to the electronic supplementary material.

Supplementary Material 1

## Data Availability

The datasets generated during the TKPSP project will not be made publicly available due to ethical reasons but will be available from the corresponding author on reasonable request. Data sharing is not applicable to this protocol article as no datasets were generated or analysed during the current study.

## Declarations

### Trial Status

The trial was scheduled to start on 4th of May 2023, and is expected to be completed by 31st January 2024.
